# Evaluation of the Activity of 4‑Quinolones against Multi-Life Stages of *Plasmodium* spp.

**DOI:** 10.1021/acsomega.5c08663

**Published:** 2025-11-05

**Authors:** Yasmin Annunciato, Everton M. da Silva, Jéssica E. Araújo, Leandro do N. Martinez, Sofia Santana, Eyob A. Workneh, Luís C. S. Alvarez, Marcela L. Magalhães, Alice Oliveira Andrade, Wallyson de J. da Costa, Guilherme Campolina, Matheus Nascimento Santana, Camila S. Barbosa, Erica P. M. L. Peres, Caio S. Moura, Najara A. C. dos Santos, Alessandra da S. Bastos, Roberto Rudge de M. Barros, Marcos L. Gazarini, Jansen Fernandes Medeiros, Carolina B. G. Teles, Fabio T. M. Costa, Ana C. Alves, Dhelio B. Pereira, João Pinto, Pedro V. L. Cravo, Rafael Victorio Carvalho Guido, Miguel Prudêncio, Maisa da S. Araujo, Arlene G. Corrêa, Gustavo C. Cassiano, Anna C. C. Aguiar

**Affiliations:** 1 Laboratory of cell biology and biochemistry of parasitic diseases, Biosciences department, 28105 Universidade Federal de São Paulo, Rua Silva Jardim, 136 - Vila Matias, Santos, São Paulo 11015-020, Brazil; 2 Department of Chemistry, 67828 Universidade Federal de São Carlos, Rodovia Washington Luís, s/n, São Carlos, São Paulo 13565-905, Brazil; 3 Malaria Vector Production and Infection Platform (PIVEM)/Entomology Lab, Fundação Oswaldo Cruz, R. da Beira, 7671 − Lagoa, Porto Velho, Rondônia 76812-245, Brazil; 4 Bioassay Platform for Malaria and Leishmaniasis, Fundação Oswaldo Cruz, R. da Beira, 7671 − Lagoa, Porto Velho, Rondônia 76812-245, Brazil; 5 Plasmodium infection and anti-malarial interventions, GIMM - Gulbenkian Institute for Molecular Medicine, Avenida Prof. Egas Moniz, Lisboa 1649-035, Portugal; 6 Laboratory of Tropical Diseases Prof. Luiz Jacintho da Silva, Department of Genetics, Evolution, Microbiology and Immunology, University of Campinas (UNICAMP), Cidade Universitária Zeferino Vaz - Barão Geraldo, Campinas, São Paulo 13083-970, Brazil; 7 Laboratory of Malaria Research, Microbiology and Imunology Departament, 28105 Universidade Federal de São Paulo, Rua Botucatu, 862 - Vila Clementino, São Paulo, São Paulo 04023-062, Brazil; 8 Global Health and Tropical Medicine, Associate Laboratory in Translation and Innovation Towards Global Health, Instituto de Higiene e Medicina Tropical, Universidade Nova de Lisboa, Rua da Junqueira, 100, Lisbon 1349-008, Portugal; 9 Medicina Tropical e Doenças Infecciosas Department, Centro de Pesquisa em Medicina Tropical de Rondônia - CEPEM, Av. Guaporé − Lagoa, 415 303, Porto Velho, Rondônia 76.812-329, Brazil; 10 São Carlos Institute of Physics, Universidade de São Paulo, Avenida Trab. São Carlense, 400 - Parque Arnold Schimidt, São Carlos, São Paulo 13566-590, Brazil; 11 Faculdade de Medicina da Universidade de Lisboa, Acadêmia de Ciências da Faculdade de Medicina, Av. Prof. Egas Moniz MB, Lisboa 1649-028, Portugal

## Abstract

Malaria is a critical global health problem, with high mortality and morbidity rates, which ultimately hinder socioeconomic development in endemic areas. The evolution of drug resistance in malaria parasites, particularly in the case of *Plasmodium falciparum*, and the scarcity of effective drugs both for case management and for blocking transmission contribute to the aggravating malaria's burden. The present study sought to evaluate the potential of novel 4-quinolone derivatives as multistage antimalarials with predicted activity against asexual intraerythrocytic, liver, and transmission-blocking stages. We show that compound 1, a natural 2-substituted-4-quinolone, previously isolated from plants and microorganisms, and its derivatives **2**–**6**, displayed significant activity against *Plasmodium* sexual stages in both *ex vivo* and *in vivo* assays and effectively prevented hepatic cell infections, with compound **1** displaying the highest activity against both sexual and asexual stages. Collectively, we conclude that these compounds warrant further studies toward the development of new antimalarial drugs.

## Graphical abstract

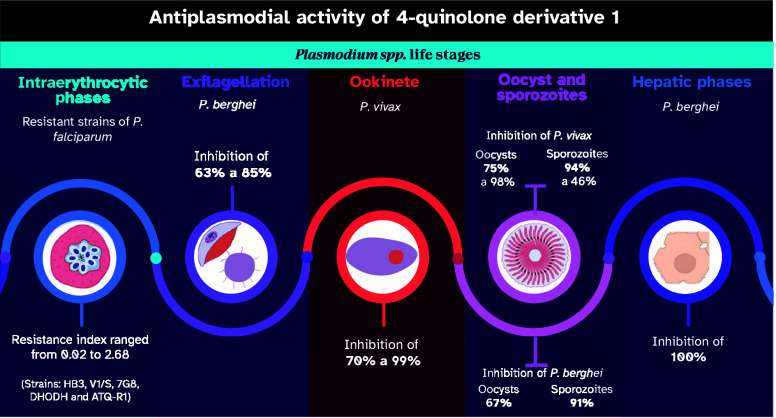

## Introduction

Malaria remains a significant public health issue, particularly affecting tropical and developing countries.^1^ The disease is caused by *Plasmodium* spp., which are obligatory intracellular parasites of the phylum Apicomplexa. *Plasmodium* parasites have a complex life cycle involving multiple stages in both female *Anopheles* mosquitoes and humans.^2^ Among the species of *Plasmodium* that affect humans, *P. falciparum* causes the most severe form of malaria,^3^ while *P. vivax*, once considered benign, is now recognized to also cause severe complications and even death.^4^

When an infected mosquito bites a mammalian host, *Plasmodium* sporozoites are injected into the bloodstream and migrate to the liver, where they then reproduce asexually. This leads to the release of thousands of merozoites into the bloodstream, which invade red blood cells, multiply, and eventually cause the typical symptoms of malaria. Some merozoites differentiate into gametocytes, which are taken up by mosquitoes during blood meals. Inside the mosquito, gametocytes undergo sexual reproduction, ultimately producing sporozoites that can infect mammals.^5^

Most malaria drugs currently used for treatment mainly act on the intraerythrocytic stage, which is associated with clinical symptoms (Target Candidate Profile 1 - TCP-1). Drugs that affect the hepatic stage (TCP-4) may be used for prophylaxis, while those targeting the parasite’s sexual forms (TCP-5) have the potential to interrupt transmission.^6,7^ The cytochrome bc1 complex is vital to the electron transport chain (ETC) and plays a key role in pyrimidine biosynthesis.^8^ At present, atovaquone (ATQ) is the clinical drug targeting the *P. falciparum* bc1 complex. Combined with proguanil, it is used for the curative and prophylactic treatment of malaria.^8^ Some strains of *P. falciparum* resistant to ATQ display a Y268S mutation in cytochrome bc1 complex^9^ and are unable to develop within mosquitoes. Although ATQ does not display inhibitory activity over mature gametocytes of *P. falciparum*,^10,11^ its transmission-blocking potential, by acting against ookinetes, oocysts, and sporozoites of *P. berghei* is well-acknowledged.^12^ As such, efforts toward finding drugs that inhibit the bc1 complex constitute an attractive strategy for developing new drugs with multistage antiplasmodial action.

ATQ monotherapy quickly induces resistance, justifying research into novel second-generation *P. falciparum* bc1 inhibitors. This includes the development of new 4-quinolones with significant activity against resistant *P. falciparum* strains, including those resistant to ATQ.^13^ Moreover, these compounds were shown to be slow-acting inhibitors of the asexual blood stages, with some of them showing submicromolar inhibitory activities (IC_50_s ranged from 100 to 0.15 μM).^13^ Importantly, the most promising compounds displayed high selectivity indices (e.g., SI > 120 for compound 1), reflecting a specificity for *Plasmodium* over mammalian cells, thus minimizing the risk of host toxicity.^13^

Herein, we further explored the potential activity of these natural 4-quinolone derivatives by investigating their transmission-blocking potential activity. In this sense, we investigated the multistage potential of the quinolone natural compound (**1)** and its derivatives **2–6** (Figure 1). Using a comprehensive approach, including *in vitro* sexual-stage cultivation, *ex vivo* assays with *P. vivax* isolates from the Brazilian Amazon, and *in vivo* mosquito infection models, we provide the first experimental evidence that these compounds can block malaria transmission.

**Figure 1:**
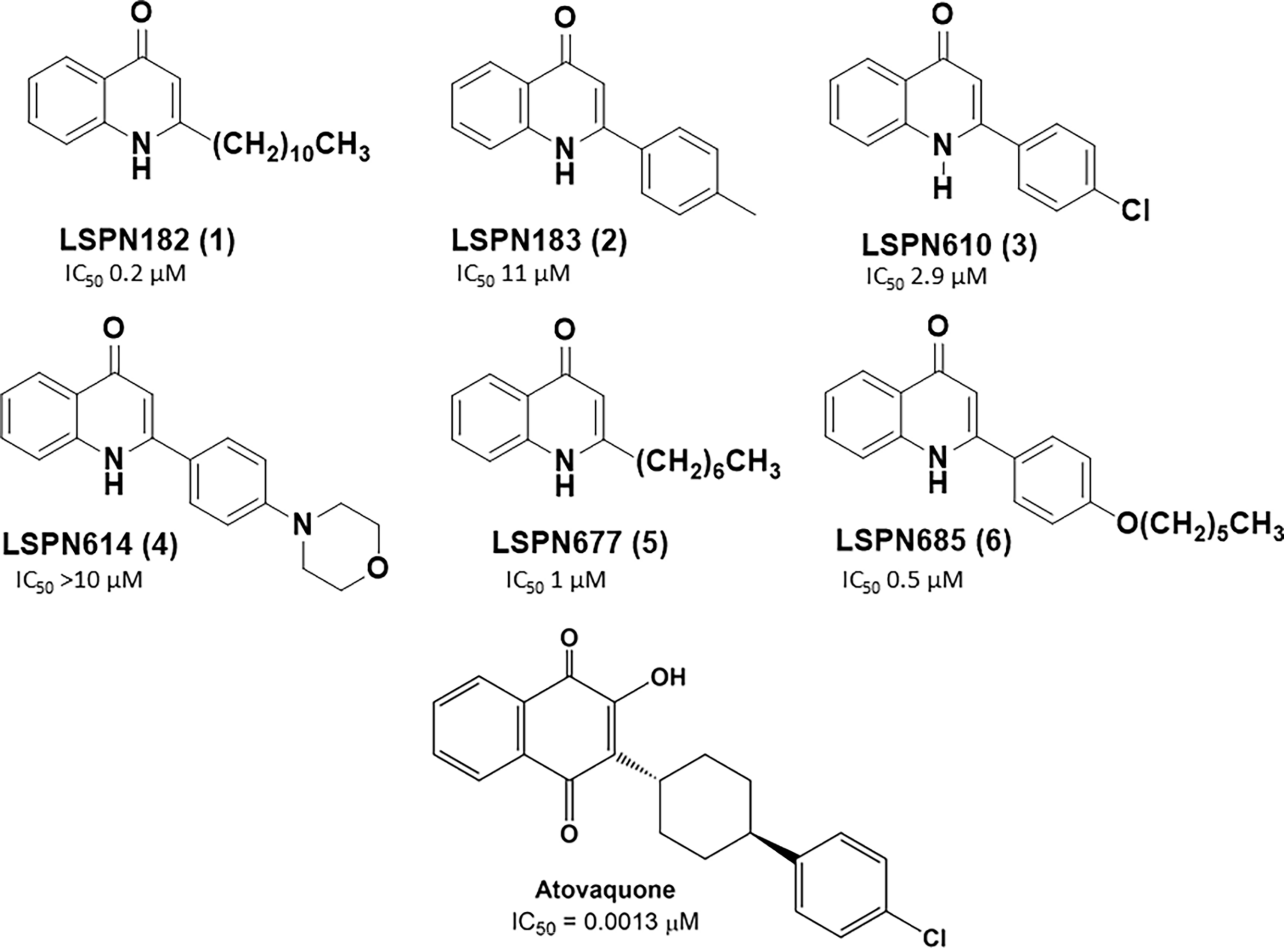
Chemical structure of 4-quinolone derivatives **1**-**6** and atovaquone, as a control. The IC_50_ value of each compound was calculated based on the antiplasmodial activity against the asexual blood stages of *Plasmodium falciparum* 3D7 sensitive strain.

## Results

### Antiplasmodial Evaluation of Resistant Strains of *P. falciparum* Asexual Stages

Previous studies demonstrated that 4-quinolone compounds effectively inhibited the bc1 complex in enzymatic assays.^13^ These compounds showed potent activity against the *P. falciparum* TM90C6B (resistance index **>**769)^13^ strain, which carries the Y268S mutation at the Qo site of the cytochrome bc1 complex, (Method S1, Result S1, and Figure S8) and exhibits over 3,000-fold resistance to ATQ.^13,14^ To further investigate the inhibitory properties of the 4-quinolone series, a representative panel of *P. falciparum* multidrug-resistant strains was selected. As compound **1** showed the best antiplasmodial activity against the asexual phases in a work previously published by Souza et al.,^13^ it was chosen to be tested against the HB3, V1/S, 7G8, SB1-A6, DHODH, and ATQ_R1 ^15^ drug-resistant strains and the sensitive 3D7 strain. HB3 is resistant to pyrimethamine, as are the other strains. The V1/S strain exhibits resistance to chloroquine and quinine. 7G8 is resistant to chloroquine and piperaquine. ATQ_R1 ^15^ is resistant to ATQ (point mutation in cytochrome bc1 complex – V259L) and chloroquine, and SB1-A6 is highly resistant to ATQ, but there is no evidence that this strain exhibits resistance to ATQ at the level of the cytochrome bc1 complex. No evidence of cross-resistance was identified (Figure S1 and Table 1) for the HB3, V1S, DHODH, 7G8, and ATQ_R1 strains, with IC_50_ values varying from 0.06 to 0.8 μM, and the resistance index ranging from 0.3 to 4. By contrast, a considerable resistance index (RI = 35) was observed for the SB1-A6 strain.

**Table 1:** *In Vitro* Resistance Indexes of Compound **1**, Based on the Ratio of IC_50_ Values between Multidrug-Resistant and 3D7-Sensitive *P. falciparum* strain

	**Strains (IC**_**50**_ **μM)** ^a^						
	**3D7**	**HB3**	**V1S**	**7G8**	**SB1-A6**	**ATQ_R1**	**DHODH**
**1**	0.2 ± 0.1	0.43 ± 0.05	0.80 ± 0.1	0.27 ± 0.01	7 ± 1	0.6 ± 0.1	0.06 ± 0.01
**RI**		2.1	4.0	1.3	35	3.0	0.3

a Data represents the average and SD values of at least three independent assays.

### Transmission-Blocking Activity of the 4-Quinolone Series

#### *In Vitro* Gametocidal Activity against *P. falciparum*

We assessed the transmission-blocking activity of the 4-quinolones by testing the compounds *in vitro* against late-stage gametocytes (IV and V counted together) of *P. falciparum*, using a bioluminescence assay with the luciferase-expressing NF54 strain. Compounds **1**, **5,** and **6** were selected as representative of the series based on their high *in vitro* activity against asexual phases of *P. falciparum* (IC_50_s of 0.21 ± 0.04; 1.0 ± 0.2 and 0.56 ± 0.07 μM, respectively),^13^ and showed moderate gametocyte inhibitory activity after 48 h of incubation at 20 μM. Compound **6** exhibited the highest inhibition (68%) (Table 2, Table S1). Additionally, all compounds were tested at a final concentration of 1 μM, but no inhibition was observed.

**Table 2:** *P. falciparum* Gametocyte Inhibition by Compounds **1**, **5,** and **6** (Concentration of 20 μM)

**Compound [20 μM]**	*Pf* **gametocytes Inhibition (%)** ^a^
**1**	44 ± 4
**5**	25 ± 4
**6**	68 ± 25
**MB** ^b^	100 ± 0

a Data represents the average and SD values of at least three independent assays.

b Methylene blue (MB) was used as a control at a concentration of 5 μM.

#### *Ex Vivo* Activity against *P. vivax* Ookinetes

We assessed the ability of compounds **1**–**6** to inhibit the *in vitro* formation of *P. vivax* ookinetes. For this, we collected samples from *P. vivax* monoinfected patients (n = 6) carrying gametocytes in the bloodstream and incubated them for 24 h in the presence of the compounds at 10 μM. All compounds inhibited at least 50% of ookinete formation, with compound **1** showing the highest activity, with a median inhibition of 97%. The control ATQ exhibited an inhibition of 97% (Figure 2 **A and** [Fig fig2] **B**). Next, compounds **1** and **6** were tested at concentrations of 2 and 0.4 μM. These compounds were selected based on the most potent activity against *P. vivax* ookinete and *P. falciparum* gametocytes, respectively. Compound **1** displayed median inhibition rates of 84% and 70% at 2 and 0.4 μM, respectively. Compound **6** showed 83% inhibitory activity when tested at a concentration of 2 μM, but showed no activity at 0.4 μM (Figure 2B).

**Figure 2:**
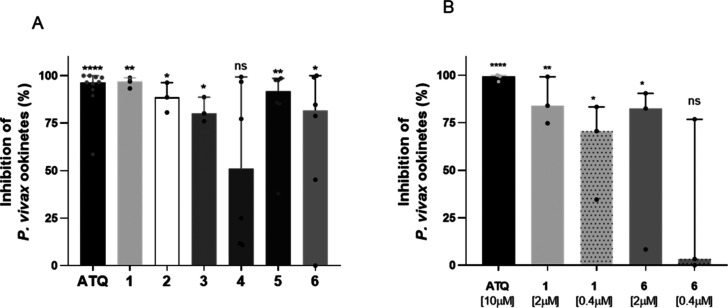
*P. vivax* ookinetes inhibition assay with quinolone-derived compounds and the control atovaquone tested at 10 μM (*p* = <0.01). A total of six independent assays was performed (A). Compounds **1** and **6** were tested at 2 μM and 0.4 μM. ATQ was used as a control. Statistical significance was determined by the Mann–Whitney test. **** < 0.0001; **0.0035 to 0.016; *0.2 to 0.4; ns = non significant (B). Each point represents a patient (*n* = 6) for 6 independent assays. Average of ookinetes/μL. *p* < 0.01. Statistical significance was determined by Mann–Whitney test. **** *p* < 0.0001; ** *p* 0.002 to 0.01; * *p* 0.02 to 0.03; ns = nonsignificant.

#### Evaluation of the Inhibitory Effect of 4-Quinolones against the Sexual Development of *P. vivax* Forms

Direct membrane feeding assays (DMFA) are fundamental for determining the efficacy of transmission-blocking intervention (TBI) candidates, as they more closely mimic the life cycle of the parasite in the mosquito vector.^12^ For this reason, we evaluated compounds **1** and **6** in DMFA using gametocytes collected from patients with *P. vivax* and laboratory-reared *A. darlingi* mosquitoes.^16^ Six patients were enrolled in our study, and 30 mosquitoes per group were dissected. Compound **1** was tested at 10 and 2 μM and caused a 95% and 74% reduction in the oocyst density per mosquito midgut (Figure 3A and Figure S2), respectively, whereas the infection prevalence was reduced by 71% and 31%, respectively (Figure 3C). The reduction of the number of oocysts in representative individual assays is shown in Figures S3 and S4. Compound **6** caused a 53% and 29% reduction in the oocyst density at 10 and 2 μM, respectively. After 14 days of mosquito feeding, the presence of sporozoites was evaluated. Compound **1** reduced the density of sporozoites per mosquito by 96% and 39% at 10 and 2 μM, respectively. Compound **6** caused a reduction of 10% and 31% in the sporozoite density at concentrations of 10 and 2 μM, respectively (Figure 3B).

**Figure 3:**
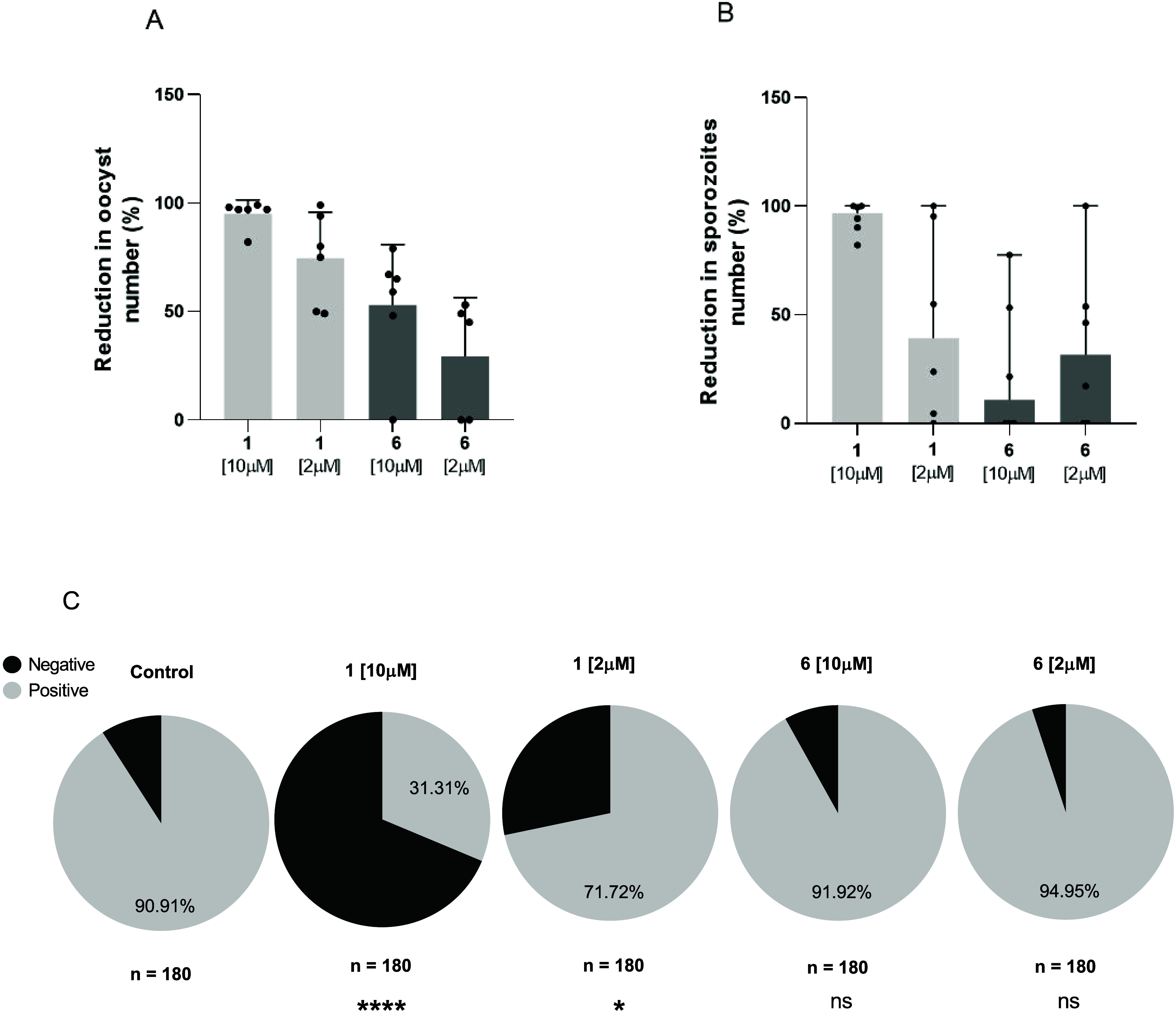
Blocking-transmission activity in *Plasmodium vivax* oocysts, calculated relative to the untreated control, for compounds **1** and **6** tested at 10 μM and 2 μM (*n* = 6 patients for 6 independent assays) (A). The reduction in sporozoite number (%) in the presence of compounds **1** and **6** at 10 μM and 2 μM (B). Prevalence of infected mosquitoes in the groups treated with **1** and **6** tested at 10 μM and at 2 μM, respectively (C). A total of 180 mosquitoes were dissected, 30 per each group (*n* = 6 groups). The blood of 6 volunteers was used in independent assays. Statistical significance was determined by Mann–Whitney test. ****p < 0.0001; * *p* = 0.04; ns = nonsignificant.

#### Assessment of *In Vivo* Transmission-Blocking Efficacy of Compound 1

Compounds **1–6** were tested at 10 μM for their ability to inhibit the formation of *P. vivax* ookinetes *ex vivo* using samples from six monoinfected patients in independent assays. During the analysis of the transmission-blocking efficacy in the *in vivo* assays with *P. berghei*, only compound **1** was tested at a concentration of 50 mg/kg x 2/12 showed ≥ 50% inhibition, whereas the positive control drug Primaquine achieved 100% inhibition (Figure 4A; Table S2, and Figure S6). Twenty-one days postinfection, the salivary glands of the mosquitoes were dissected, and 91% reduction in the infection rate and 70% reduction in the number of sporozoites were observed. Mosquitoes that fed on primaquine-treated mice were not infected (Figure 4B and 4 **E**).

**Figure 4:**
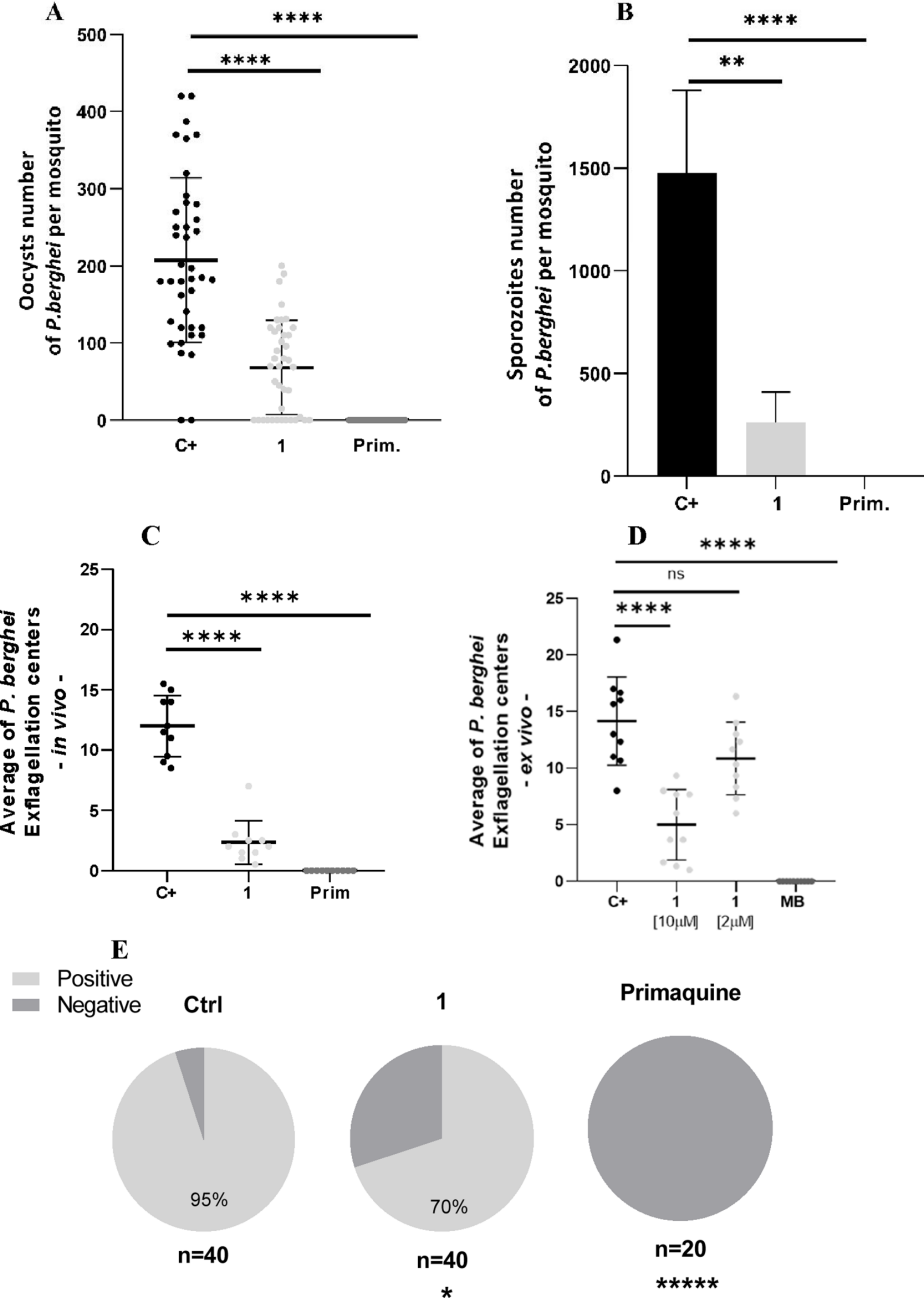
*P. berghei* oocyst per mosquito after treatment (50 mg/kg x 2/12h) with compound **1** and primaquine (25 mg/kg x 2/12h) control in the *in vivo* assay (*n* = 6 mice per group for 2 independent assays). Statistical significance between the control, compound **1** and Primaquine was determined by the Mann–Whitney test. **** *p* < 0.001 (A). Quantification of *P. berghei* sporozoite per mosquito after treatment with compound **1** (2 x 50 mg/kg, dosesd12h apart) or primaquine (25 mg/kg x 2/12h) control in the *in vivo* assay. Statistical significance was determined by the Mann–Whitney test. **p = 0.02; **** *p* < 0.001 (*n* = 6 mice per group for 2 independent assays) (B). Evaluation of the exflagellation process of *Plasmodium berghei* under treatment with compound **1** and the primaquine control in the *in vivo* assay after 12 h of treatment. Statistical significance was determined by Mann–Whitney test. **** *p* < 0.001 (*n* = 6 mice per group) (*n* = 6 mice per group for 2 independent assays) (C). Exflagellation centers in the group treated with compound **1** and the methylene blue control in the *ex vivo* assay (*n* = 3 animals per group for 3 independent assays). Statistical significance was determined by Mann–Whitney test. ****p < 0.001; ns = nonsignificant **(D**). Prevalence of infection in *An. stephensi* mosquitoes infected with *P. berghei* in the groups treated with compound 1 and in the control. Statistical significance was determined by Mann–Whitney test. * *p* = 0.0375, **** *p* < 0.001; ns = nonsignificant. The ‘n’ number of each group represents the number of mosquitoes (E).

The impact of compound **1** on *P. berghei* fertilization and development in mosquitoes was evaluated through *in vivo* and *ex vivo* assays. At mosquito feeding, blood samples showed an 85% reduction in male exflagellation centers after treatment (Figure 4C). *Ex vivo* incubation for 15 min with compound **1** at 10 and 1 μM reduced the exflagellation centers by 64% and 24%, respectively. Methylene blue, the positive control drug, completely inhibited the formation of exflagellation centers at 5 μM (Figure 4D; Table S2 and Figure S5).

#### *In Vitro* Activity against the Hepatic Stage of *P. berghei*

The efficacy of 4-quinolone compounds against *P. berghei* hepatic stages was assessed using a bioluminescence method to quantify parasite loads in Huh-7 cells infected with luciferase-expressing sporozoites in presence of different compound concentrations. Except for compound **2**, the IC_50_ values of all tested compounds were in the nanomolar range, with compound **1** being the most potent inhibitor, showing an IC_50_ value of 250 nM (Table 3, Figure S7). Furthermore, the Alamar Blue cytotoxicity analysis of cell confluency indicated that none of the tested concentrations were harmful to the host cells at the concentrations employed in our assays.

**Table 3:** Effect of 4-Quinolone Derivatives (IC_50_) on Hepatoma Cell Confluency (%) and the Hepatic Stages of *Plasmodium berghei*

**Compound**	**IC**_**50**_ **(μM)** *Pb* **liver stages**	**Confluency% [10 μM]**	**Confluency % [1 μM]**
**1**	0.25 ± 0.01	79 ± 2	97 ± 6
**2**	1.3 ± 0.2	104 ± 4	163 ± 14
**3**	0.64 ± 0.04	140 ± 1	147 ± 1
**5**	0.30 ± 0.01	147 ± 3	100 ± 1
**6**	0.38 ± 0.04	102 ± 11	105 ± 3
**Primaquine** ^a^		104.0 ± 0.2	109 ± 10

a Primaquine was used as the positive control of the cytotoxicity assays.

#### Mitochondrial Assessment of Compound 1 in HepG2 Cells Cultured in Glucose or Galactose

To evaluate the mitochondrial activity of compound **1**, we employed a galactose-conditioning assay using HepG2 cells. This method distinguishes mitochondrial-dependent toxicity by comparing cell viability in media containing either glucose or galactose as the primary carbon source. HepG2 cells treated with compound **1** exhibited strong growth inhibition under galactose conditions, with cell viability falling below 0.5% across all tested concentrations (3.125–50 μM). In contrast, cells maintained in glucose-containing media showed over 50% viability, indicating a clear dependence on mitochondrial metabolism for compound-induced toxicity. ATQ, used as a mitochondrial toxicity control, did not reduce cell survival below 60% under either condition. These results suggest that compound **1** disrupts mitochondrial function and highlights the importance of mitochondrial metabolism in its cytotoxic profile (Figure 5 **)**.

**Figure 5:**
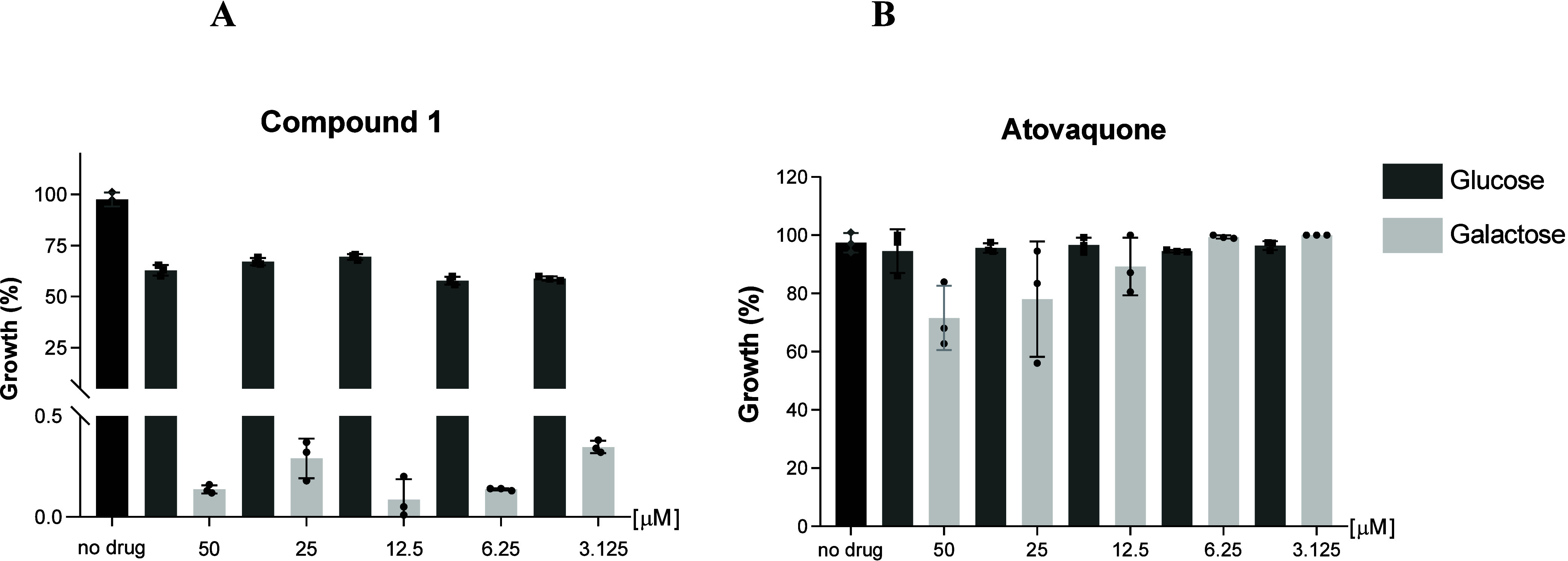
Viability of HepG2 cells exposed to Compound **1 (A)**, and the cells exposed to atovaquone (B) across five concentrations: 3.125, 6.25, 12.5, 25, and 50 μM. Dark gray bars represent cells cultured in glucose, light gray bars represent cells cultured in galactose, and black bars represent untreated control cells (100% viability).

## Discussion

Parasite resistance to antimalarial drugs, along with their side effects and contraindications, has increased the need for discovering new molecules, including drugs able to block malaria transmission.^17^ Investigations into 4-quinolones have demonstrated that this class of compounds possesses a compelling antiplasmodial profile ^20;21^. These include endochin derivatives with substitutions at the 3-position of the 4-quinolone, such as the endochin analogue ELQ-300,^18^ as well as quinolones with substituents at the C2-position, such as the compounds investigated in this work^19–21^ (Figure 1 and Table 4).

**Table 4:** Examples of 4­(1H)-quinolones with Antiplasmodial Activity^a^

**4(1H)-quinolones**	**Compound**	**Strain**	**IC**_**50**_ **[nM]**
3-substituted	ELQ-121 ^18^	TM90C2B	1.7
3-substituted HDQ derivatives	CK-2–68 ^20^	3D7	31
3-carboxyl	Decoquinate^22^	164/GFP	10

a ELQ-121,^18^ a 3-substituted 4­(1H)-quinolone; CK-2-68,^20^ a 3-substituted 4­(1H)-quinolone HDQ derivative and Decoquinate,^22^ a 3-carboxyl 4­(1H) quinolone.

The 4-quinolones act by inhibiting the mitochondrial electron transport chain at the cytochrome bc1 complex (complex III). This disruption impairs electron transfer and subsequently affects the activity of the dihydroorotate dehydrogenase (DHODH) enzyme.^23^ DHODH is crucial for pyrimidine synthesis and essential for *P. falciparum* intraerythrocytic development. Compound **1** demonstrated potent activity against a DHODH resistant mutant (generated by our research group through drug pressure with DSM265).^15^ Previous studies revealed that the 4-quinolone compounds studied herein are potent and selective inhibitors of the cytochrome bc1 complex, with a selectivity index (SI) > 120.^13^ The *P. falciparum* strain SB1-A6 accumulates both a copy number variation and a specific mutation in *Pf*DHODH, and these genetic polymorphisms contribute to the pan-resistant phenotype,^24^ likely accounting for the absence of inhibitory activity of 4-quinolones against it. SB1-A6 retains the sensitivity profile of its D6 parent to chloroquine, quinine, pyrimethamine, and 5-fluoroborate.^24^ Nonetheless, we note that compound **1** showed submicromolar activity (ranging from 290 to 790 nM) with a resistance index no greater than 2.7, even against the atovaquone-resistant strains (ATQ_R1 and previously tested TM90C6B^13^). These results suggest that although these compounds are able to inhibit the cytochrome bc1 complex, they are not directly associated with the same binding target as ATQ. From a molecular perspective, and in agreement with previous work,^16^ it is interesting to note that the molecule containing alkyl substituents at position 2, such as compound **1**, with an addition of 11 carbons, showed greater efficacy in inhibiting the *P. falciparum* 3D7 strain asexual stage (IC_50_ = 0.21 μM). Conversely, a structural modification at position 2 with a shorter alkyl chain decreased the inhibitory efficacy, as seen for compound **5**, which had an heptyl alkyl-chain and presented an IC_50_ value of 1 μM Although compound 1 with a long alkyl chain at the 2-position showed superior potency, we also investigated aromatic substituents at this position to expand the structure–activity relationship, explore potential improvements in pharmacological and transmission-blocking properties, and to assess how different steric and electronic features could impact the multistage antiplasmodial profile of 4-quinolones.

Previously, in a different work by Sáenz et al.,^25^ 4-quinolone derivatives with substituents at positions 2, 3, 6, and 7 were tested for their transmission-blocking activity, particularly for compounds P4Q, PEQ, and THA,^25^ and were shown to significantly reduce the number of infected *An. freeborni* mosquitoes and oocysts in the midgut when *P. falciparum* gametocytes were exposed to the drugs.^25^ We observed similar results when *An. darlingi* and *An. stephensi* mosquitoes were treated with compound **1**, leading to a substantial reduction in infection. Subsequently, the number of oocysts in mosquito midguts was counted, and early stage gametocyte treatments with THA-93, ICI 56.780 and P4Q-146 led to a 100% reduction in oocyst numbers, while P4Q-95 and P4Q-105 achieved a 99% reduction. Our study demonstrated that 4-quinolones **1** and **6** also showed strong oocyst reduction in *P. vivax* infections, with compound **1** reducing the oocyst numbers by 98% at its highest concentration. Collectively, these results underscore the potential of 4-quinolone derivatives in blocking malaria transmission.

While we observed significant reductions in mosquito infection, oocyst numbers, and sporozoites, the study by Sáenz et al.^25^ suggests that specific substitutions at positions 2, 3, 6, and 7 might be more efficient at blocking transmission. This is noteworthy since our 4-quinolone derivatives have a modification at position 2, which may influence their transmission-blocking activity. Further exploration of these scaffolds and their substituents may contribute to optimizing antimalarial activity.

Compound **1** demonstrated significant inhibitory activity against *P. vivax* oocyst development at concentrations below 10 μM, highlighting its strong transmission-blocking potential. Comparatively, several approved compounds, such as endoperoxides, lumefantrine, halofantrine, and mefloquine, required concentrations at around 10 μM to inhibit oocyst development,^25^ albeit these studies primarily focused on *P. falciparum*, with limited data being available regarding efficacy assays against *P. vivax* oocyst formation ^27;28^. Other studies have also shown that many antimalarials fail to significantly inhibit oocyst development or require much higher concentrations for efficacy,^13,20,24,25^ further re-enforcing the value of the 4-quinolone derivatives reported herein, particularly against *P. vivax*. Furthermore, the short incubation period of DMFA assays suggests that compounds need to act swiftly during the fertilization phase rather than killing gametocytes. In this context, the 4-quinolones derivatives **1** and **6** represent a promising approach for interrupting malaria transmission, particularly by targeting early stages in the mosquito vector. This is evidenced by the high inhibition rate (98%) of *P. vivax* ookinetes demonstrated by **1**. We also note that, to our knowledge, this is the first report evaluating the efficacy of 4-quinolone derivatives against the sexual stages of *P. vivax*, further highlighting the potential of this chemical class as a novel transmission-blocking strategy.

The sporozonticidal activity in mosquitoes infected with *P. falciparum* was assessed by feeding mosquitoes with drug-treated blood at a concentration of 1 μM.^20^ Sáenz et al., reported that only the ICI 56.780 compound significantly reduced salivary gland infections by 80% compared to the untreated control group. In our study, compound **1** showed a sporozoite reduction greater than 80% at 10 μM, suggesting that its structural modification with the extended alkyl chain contributed to blocking disease transmission at this stage of the cycle.^25^

Biagini et al.^20^ also evaluated the activity of quinolone derivatives with a modification at position 2 for their transmission-blocking potential. None of the inhibitors demonstrated activity against *P. falciparum* gametocytes in late stages IV and V. In our study, compound **6** showed an inhibition of 64% against *P. falciparum* gametocytes in late stages IV and V. This finding suggests that the inhibitory effect of 4-quinolone derivatives is likely associated with the fertilization process between microgametocytes and macrogametocytes. In addition, the compounds evaluated by Biagini et al.^20^ exhibited remarkable activity against the production of *P. berghei* ookinetes (IC_50_s of 73 nM and 154 nM, respectively). Similarly, compound **1** showed a moderate reduction in the number of exflagellation centers, oocysts, and sporozoites of *P. berghei* in an *in vivo* transmission-blocking assay (85%, 67%, and 91%, respectively).

It is known that the asexual stages display mitochondria with tubule-like cristae, while the gametocyte mitochondria have a greater number of cristae, including densely packed tubular cristae.^26–29^ These variations indicate that gametocytes possess more metabolically active mitochondria, which may be essential for survival during transmission.^26^ Biochemical evidence indicates heightened mitochondrial activity in gametocytes and mosquito stages of the malaria parasite. In *P. berghei,* complex II is crucial for oocyst formation, suggesting a metabolic shift from glycolysis to oxidative phosphorylation in mosquitoes. Deactivation of NDH_2_ further impedes oocyst maturation, highlighting the importance of an active mitochondrial electron transport chain during these stages.^25^ Given these observations on the inhibitory effect of 4-quinolones on transmission stages, our findings support the assumption that mitochondrial function is crucial during the sexual phases of *Plasmodium* sp., thereby reinforcing the validity of mitochondria as a primary target for transmission-blocking drugs.

In our study, the infection of Huh-7 hepatocytes by *P. berghei* sporozoites was evaluated in the presence of 4-quinolone derivatives, revealing significant inhibitory effects at low micromolar concentrations without hepatocyte toxicity. Similar methodologies have been applied in other studies, such as the evaluation of 25 quinolone derivatives against hepatic stages of *P. yoelii* in mouse hepatocytes. In this context, five derivatives, including grepafloxacin, exhibited notable activity against hepatic schizonts, with IC_50_ values ranging from 4.4 to 36.3 μg/mL.^27^ Grepafloxacin and trovafloxacin showed potent activity against *P. falciparum* hepatic stages, with IC_50_ values of 5.0 ± 0.2 and 21.6 ± 2.6 μg/mL, respectively, highlighting their potential for clinical application.^20,26^

Marroquin et al.^28^ showed that HepG2 cells cultured in galactose exhibit increased oxygen consumption and enhanced sensitivity to mitochondrial inhibitors, such as antimycin A, a BC1 complex (complex III) blocker. Compound **1** induced mitochondrial toxicity only in HepG2 cells grown in galactose, suggesting that it interferes with oxidative phosphorylation. These findings highlight the importance of using galactose-cultured HepG2 cells to reveal mitochondrial toxicities that are not detectable under glycolytic conditions and also reinforce the action of this class of compounds on the bc1 complex. Our findings align with these results, particularly in the case of **1**, which shares a similar profile of high efficacy and low toxicity. In summary, the activity against multiple life cycle stages of the parasite, including asexual blood stages, liver stages, and transmission-blocking activity, is well in line with MMV’s vision for next-generation antimalarials that can contribute to both treatment and eradication strategies by targeting more than one stage of the parasite life cycle.^6,7^

## Methods

### Synthesis of the 4-Quinolone Derivatives

The commercial reagents were purchased from Sigma-Aldrich. The products were purified through a chromatographic column using silica gel 60, 230–400 mesh. TLC was performed on silica gel 60 F254 supported on aluminum sheets. Reactions were irradiated in a focused microwave oven CEM Discover (Matthews, NC - USA). Melting points were obtained on a Buchi M-560. ^1^H and ^13^C NMR spectrum were recorded on a Bruker DRX 400 MHz spectrometer. Chemical shifts (δ) were presented in ppm units and the coupling constants (J) in Hertz (Hz). Signals multiplicities were expressed by the following abbreviations: s (singlet), d (doublet), t (triplet), q (quartet), and m (multiplet). The exact mass measurement was carried out using a micrOTOF Q IITOF Mass Spectrometer (Bruker Daltonics, Billerica, MA, USA) equipped with an ESI ion source (positive ionization mode).

The 2-undecyl-4­(1*H*)-quinolone (**1**) was previously reported to possess antiplasmodial activity (0.2 μM).^13^ The synthesis of 2-substituted-4-quinolones **1**-**6** was performed via cyclization of the corresponding acylated 2-aminoacetophenone under microwave irradiation, as previously reported by our research group^13,29^ (Scheme 1).

**Scheme 1:**
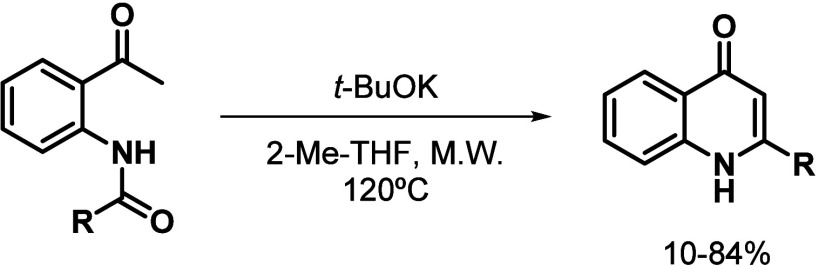
Synthesis of 2-Substituted-4-quinolones **1**-**6**

#### 2-Undecilquinolin-4­(1H)-one (**1**):^13^

55% yield. IR (ν_max,_ KBr): 756, 765, 1246, 1356, 1365, 1441, 1489, 1552, 1593, 1657, 1713, 2331, 2358, 2850, 2870, 2920, 2954 cm^–1^. ^1^H NMR (200 MHz, CD_3_OD) δ: 0.86 (t, 3H, *J* 6.5 Hz); 1.22–1.52 (m, 16H); 1.71 (qui, 2H, *J* 7.8 Hz); 2.69 (t, 2H, *J* 7.9 Hz); 6.19 (s, 1H); 7.35–7.41 (m, 1H); 7.66–7.70 (m, 2H); 8.11 (d, 1H, *J* 8.0 Hz). ^13^C NMR (50 MHz, CD_3_OD) δ: 14.1; 22.7; 25.6; 26.9; 28.7; 28.8; 29.4; 29.6; 29.7; 31.7; 34.9; 107.6; 118.13; 123.8; 124.5; 124.7; 131.6; 139.3; 156.7; 179.3. MS (*m/z)*: 274 (M^+^), 177, 162, 135, 121, 120, 106, 92, 69, 55.

#### 2-p-Tolylquinolin-4­(1H)-one (**2**):^30^

68% yield. mp 300 °C. IR (ν_max,_ KBr): 482, 514, 536, 567, 671, 756, 815, 873, 958, 1024, 1141, 1186, 1245, 1315, 1357, 1440, 1471, 1510, 1542, 1595, 1635, 1652, 1701, 2914, 2964, 3066, 3087, 3116 cm^–1^. ^1^H NMR (200 MHz, CD_3_OD) d: 2.46 (s, 3H); 6.78 (s, 1H); 7.42 (d, 2H, J 8.0 Hz); 7.52 (ddd, 1H, J 1.6, 1.7, 4.7 Hz), 7.73 (dd, 1H, J 1.7, 8.0 Hz); 7.80–7.90 (m, 2H); 8.31 (dd, 1H, J 0.8, 6.3 Hz). ^13^C NMR (50 MHz, CD_3_OD) d: 22.7, 103.7, 119.6, 123.7, 125.4, 125.6, 126.7, 127.9, 128.6, 132.8, 140.3, 141.5, 153.6, 176.4.

#### 2-(4-Chlorophenyl)-6-morpholinoquinolin-4­(1H)-one (3):^13^

38% yield. mp 317 °C. IR (ν_max,_ KBr): 3248, 3138, 2850, 1627, 1598, 1535, 956, 823, 794 cm^–1^. ^1^H NMR (DMSO-*d*_6_, 400 MHz) δ: 3.17 (t, 4H, *J* 1.8 Hz); 3.79 (t, 4H, *J* 1.8 Hz); 6.30 (s, 1H); 7.44 (bs, 1H); 7.52 (d, 1H, *J* 8.4 Hz); 7.67 (t, 3H, *J* 9.7 Hz); 7.86 (d, 2H, *J* 8.3 Hz); 11.6 (s, 1H); HRMS: calcd for C_19_H_19_ClN_2_O_2_^+^ *m*/*z* [M + H^+^] 341.1051, found 341.1053.

#### 2-(4-Morfolinofenil)­quinolin-4­(1H)-one (**4**):^13^

62% yield. mp: 319 °C. IR (ν_max,_ KBr): 2956; 1626; 1600; 1593; 1121; 925 cm^–1^. ^1^H NMR (DMSO-*d*_6_, 400 MHz) δ: 11.49 (s, 1 H); 8.07 (d, 1 H, *J* 7.6 Hz); 7.76 (t, 3 H, *J* 7.6 Hz); 7.65 (t, 1 H, *J* 7.6 Hz); 7.31 (t, 1 H, *J* 7.5 Hz); 7.12 (d, 2 H, *J* 8.6 Hz); 6.31 (s, 1 H); 3.77 (t, 4 H, *J* 1.8 Hz); 3.26 (t, 4 H, *J* 1.8 Hz). ^13^C NMR (DMSO-*d*_6_, 400 MHz) δ: 185.4; 152.7; 149.9; 139.9; 131.7; 128; 123.9; 123.0; 118.1; 113.8; 105.5; 65.9; 47.4.

#### 2-Heptylquinolin-4­(1H)-one (**5**):^13^

84% yield. mp 137 °C. IR (ν_max_, KBr): 580, 752, 1321, 1355, 1442, 1475, 1505, 1550, 1595, 1639, 2856, 2924 cm^–1^. ^1^H NMR (400 MHz, DMSO-*d*_6_) δ: 0.85 (t, 3H, *J* 6.7 Hz); 1.19 – 1.39 (m, 8H); 1.65 – 1.68 (m, 2H); 2.58 (t, 2H, *J* 7.6 Hz); 5.91 (s, 1H); 7.26 (t, 1H, *J* 7.3 Hz); 7.53 (d, 1H, *J* 8.2 Hz); 7.65 – 7.55 (m, 1H); 8.02 (d, 1H, *J* 7.9 Hz); 11.5 (s, 1H). ^13^C NMR (100 MHz, DMSO-*d*_6_) δ: 13.9; 22.0; 28.3; 28.4; 31.1; 33.2; 107.6; 117.8; 122.7; 124.6; 124.7; 131.4; 140.1; 153.5; 176.8. HRMS: calcd for C_16_H_22_NO^+^ *m*/*z* [M + H^+^] 244.1696, found 244.1694.

#### 2-(4-(Hexyloxy)­phenyl)­quinolin-4­(1H)-one (**6**):^13^

Ten % yield. mp 241 °C (degraded). White solid. ^1^H NMR (400 MHz, DMSO-*d*_6_) δ 8.09 (d, *J* 8 Hz, 1H); 7.79 (d, *J* 8 Hz, 2H); 7.76 (s, 1H); 7.66 (t, *J* 8 Hz, 1H); 7.33 (t, *J* 8 Hz, 1H); 7.12 (d, *J* 8 Hz, 2H); 6.32 (s, 1 H); 4.06 (t, *J* 8 Hz, 2H); 1.76–1.72 (m, 2H); 1.45–1.41 (m, 2H); 1.32–1.31 (m, 4H); 0.90–0.88 (m, 3H). ^13^C NMR (101 MHz, DMSO-*d*_6_) δ 179.0; 176.2; 160.0; 149.3; 139.9; 131.2; 128.3; 125.4; 124.1; 122.6; 118.1; 114.3; 105.8; 67.2; 30.4; 28.0; 24.6; 21.5.

### In Vitro Cultivation and Antimalarial Evaluation of Resistant *Plasmodium falciparum*

Healthy blood samples for parasite culture were obtained from voluntary donors (approved under protocol #28176720.9.0000.0011), processed to remove white blood cells, and stored at 2 °C–8 °C for up to 15 days. Parasite strains SB1-A6, 7G8, V1/S, and HB3 were acquired from BEI-MR4, while the ATQ R1 strain, carrying a V259L gene mutation, was generated in-house. Infected erythrocytes were cultured in RPMI 1640 medium supplemented with Hepes, sodium bicarbonate, glucose, Albumax, and gentamicin, maintained at 2 °C–8 °C for up to 15 days, and incubated in a 5% CO_2_, 5% O_2_, and 90% N_2_ gas mixture at 37 °C with daily medium changes. Parasitemia was monitored using Giemsa-stained blood smears.

Bioassays were conducted with synchronized ring-stage parasites using sorbitol synchronization.^31^ Antimalarial compounds were evaluated in 96-well plates, and parasite growth was assessed using a SYBR Green-based fluorescence assay^24^ The assays were conducted when the parasite quantity reached at least 70% of the ring stage. Compounds were initially added at specific concentrations in the first wells, followed by serial dilution (1:2) ranging from 20 μM to 0.009 μM. Subsequently, a solution containing parasites in RPMI medium at a hematocrit of 2% was added to each well, and the parasites were then incubated for 72 h.

To assess parasite growth, the culture medium was replaced with a lysis solution (Trisbase: 49.31%, EDTA: 30.00%, Saponin: 0.16%, Triton: 0.02%) containing the fluorescent dye SYBR Green (Sigma). After a 30 min incubation, fluorescence readings were taken to determine the 50% inhibitory concentration (IC_50_) of parasite growth.^26^ The IC_50_ values were determined using concentration–response curves generated in GraphPad 8 software. Additionally, the resistance index was calculated by comparing IC_50_ values obtained for the sensitive 3D7 strain with those of the resistant strains.^32^

### Gametocidal Action of Compounds

For the *in vitro* culture of sexual stages of *P. falciparum* NF54 Luminescent strain, the Tripathi protocol^33^ was followed.

Parasite asexual stages were synchronized, and ring-stage parasites were incubated with a parasite-conditioned medium to induce stress and trigger gametocyte induction. After sexually committed parasites invaded new erythrocytes, N-acetylglucosamine was added to prevent further invasion and clear residual asexual parasites.

When a predominance of late stage (IV and V) gametocytes was identified and counted together,^15,34,35^ the MACS magnetic system was used for gametocyte enrichment, as previously described.^33,36^

The column content was eluted with RPMI medium into a 15 mL Falcon tube until translucent medium appeared, yielding ∼ 10 mL of eluate. The sample was centrifuged at 1500 rpm for 5 min at 37 °C, the supernatant discarded, and the pellet washed with preheated RPMI medium containing albumax. A 1 μL pellet sample was used to prepare a Giemsa-stained smear for gametocytaemia determination by counting 2000 cells. The gametocytemia for each assay were 71%, 64% and 31%. The remaining pellet was adjusted to 1 mL with RPMI medium containing 10% human serum at 37 °C. Total cell counts were performed using a Neubauer chamber with a 10X diluted sample.

Compounds **1**, **3**, **4**, **5**, and **6** (20 μM) were tested in a single-point assay (previously tested at 1 μM, but showed no activity), with methylene blue (5 μM) as a control. Each well contained 200,000 gametocytes in 150 μL without erythrocytes. After 48 h of incubation, a luciferase kit (Promega) was used to reveal the assay. Eighty microliters of supernatant were discarded, and the pellet was homogenized in the remaining content. Forty microliters of the sample were transferred to a clear 96-well plate containing lysis solution (1X), gently mixed, and moved to a white-bottomed 96-well plate. Luciferase (40 μL) was added, and luminescence was measured using a microplate reader with a 10-s integration time.

### Evaluation of the Activity of 4-Quinolone-Derived Compounds against Sexual Forms of *P. vivax*

#### Blood Collection from Patients with *Plasmodium vivax*

Participants were recruited from patients diagnosed with *P. vivax* malaria via Giemsa-stained blood smears at CEPEM in Porto Velho, Rondônia, Brazil (#28176720.9.0000.0011). Criteria included: *P. vivax* parasitemia >2000 parasites/μL, age 18–85, no severe malaria, no concomitant diseases, nonpregnant, and consent to study procedures. Approximately 10 mL of venous blood was collected into heparin-coated vials, maintained at 37 °C, and transported to PIVEM for DMFA. Malaria treatment followed Brazilian Ministry of Health guidelines and was unaffected by study participation.

#### Evaluation of the Activity of Compounds against *P. vivax* Ookinete in *Ex Vivo* Assay

After collecting infected samples (n = 6), gametocytemia quantification began with Giemsa staining, requiring at least 10 gametocytes per 200 leukocytes for the assay. Erythrocytes were washed with incomplete RPMI-1640 medium by centrifugation at 1500 rpm for 10 min to remove leukocytes. Following three washes, gametocytes were isolated using a 55% Histodenz purification gradient, diluted in RPMI medium (pH 7.2) at a 1:3 ratio relative to the sample volume. The gradient was prepared from a stock solution containing 27.6% (w/v) Histodenz in 5.0 mM Tris-HCl, 3.0 mM KCl, and 0.3 mM EDTA (pH 7.2).^37^ The sample was centrifuged once at 450 *g* for 15 min without a brake. After centrifugation, the pellet remaining at the interface was collected and washed twice at 2000 *g* for 5 min. The hematocrit was adjusted to 2% with ookinete medium supplemented with 20% human serum and 2.5% Albumax. Gametocytes were then added to the wells of 96-well plates with the compounds previously diluted at concentrations of 10–0.1 μM. atovaquone (ATQ) (10 μM)^38^ and DMSO (0.5%)^39^ were used as controls.

The parasites were incubated for 24 h at a temperature of 21–24 °C. After this period, the activity of the compounds was determined by microscopy, counting 100 fields to determine the parasitemia per μL in each sample, and counting the mature and immature ookinetes. Compounds were considered active with ≥ 70% inhibition at a concentration of 1 μM^39^

#### Mosquito Colonies and Direct Membrane Feeding Assay

*Anopheles darlingi* mosquitoes are maintained in the PIVEM insectary at Fiocruz Rondônia since 2018.^40^ Mosquitoes were raised at a temperature of 26 °C ± 1 °C and a relative humidity of 70 ± 10%, being fed a 15% honey solution.

Before the DMFA, female mosquitoes were starved of sucrose overnight. Six *P. vivax* isolates were used, and 40–100 mosquitoes per group were fed *P. vivax*-infected blood for 30 min using a Hemotek membrane feeder at 37 °C. Unfed mosquitoes were removed, and engorged ones were maintained for sporogony, receiving a 15% honey solution refreshed every 2 days. Midguts were dissected on day 7 and salivary glands on day 14 postblood feeding, to assess midgut oocyst load and salivary gland sporozoite load, respectively.

Mosquitoes were anesthetized on ice, immersed in 70% ethanol, and dissected in PBS. Midguts were stained with 0.2% mercurochrome and examined microscopically for oocysts. Salivary glands were pooled (5 mosquitoes per sample), and homogenized in 15 μL RPMI, and sporozoites were counted using a Neubauer chamber.

#### Evaluation of the Activity of Compounds 1 and 6 against *P. vivax* Ookinets in an *Ex Vivo* Assay

Following the collection of infected samples, gametocytemia quantification began using Giemsa staining, requiring at least 10 gametocytes per 200 leukocytes. Red blood cells were then washed with incomplete RPMI-1640 medium through centrifugation at 1500 rpm for 10 min to remove leukocytes. After three washes, gametocytes were isolated using a 55% Histodenz purification gradient, prepared by diluting the gradient in RPMI medium (pH 7.2) at a 1:3 ratio to the sample volume. The gradient was derived from a stock solution of 27.6% (w/v) Histodenz in 5.0 mM Tris-HCl, 3.0 mM KCl, and 0.3 mM EDTA (pH 7.2).^37^ The sample was centrifuged once at 2000 rpm for 15 min without brake. After centrifugation, the remaining pellet at the interface was collected and washed twice at 2000 rpm for 5 min. The hematocrit was adjusted to 2% with ookinets medium supplemented with 20% human serum and 2.5% Albumax. Then, gametocytes were added to each well of 96-well plates with compounds previously diluted at concentrations of 10–0.1 μM. ATQ (10 μM)^38^ and DMSO (0.5%)^41^ were used as controls. Parasites were incubated for 24 h at a temperature of 21–24 °C. After this period, compound activity was determined by microscopy counting in 100 fields to determine parasitemia per μL in each sample. Compounds with inhibition ≥ 70% at a concentration of 1 μM were considered active.^42^

#### Evaluation of Reduction of *P. berghei* Sexual Phases in *An. stephensi*

For this assay, a colony of *An. stephensi* was used. The lab-reared *An. stephensi* was kept under controlled conditions, with a temperature of 26 °C ± 1 °C, a 12/12 h light/dark cycle, a relative humidity of 70% ± 10%, and fed a 30% sugar solution as needed.

Female CD1 mice (4 to 6 weeks old) were infected by intraperitoneal inoculation (i.p.) containing 10 million erythrocytes infected with *P. berghei* expressing the GFP fluorescent protein.^42^ After 4 days, when parasites in the sexual stages were detected via Giemsa-stained blood smears, mice with similar gametocytemia and parasitemia were randomly divided into three groups of two animals each. The groups of two mices were treated orally with compound 1 at two doses of 50 mg/kg, spaced 12 h apart. Control groups received either the vehicle solution (30% DMSO + 35% RPMI medium +35% fetal bovine serum) spaced 12 h apart as a negative control or primaquine as a positive control at a concentration of 25 mg/kg, spaced 12 h apart. Due to reducing the number of animals in the trials, respecting the ethics committee and considering that primaquine administered at the dose tested is a well-established model in the literature, eliminating the total parasitic load of the mice, only 1 mouse was treated in each independent trial (n = 2 independent trials). Following the second dose, mice were anesthetized and placed in individual cages containing approximately 50 female *A. stephensi* mosquitoes, which had been starved for 24 h before the assay. Blood feeding lasted for 30 min, after which unfed mosquitoes were removed. Engorged females were maintained in an insectary at 21 ± 1 °C and 60% relative humidity, receiving a 30% glucose solution. Ten days postblood meal, 20 mosquitoes from each cage treated with compound **1** and negative control groups and 10 mosquitoes from the positive control treated with primaquine were dissected to microscopically evaluate oocysts in their midgut.^43^

A group of 10 mosquitoes was set aside to assess sporozoite development in salivary glands, following a protocol similar to the one described previously, with dissections performed 21 days postblood meal. Additionally, 10 μL of mouse blood was collected immediately after treatment with the compounds and incubated at room temperature for 15 min. Exflagellation was observed under optical microscopy to evaluate the effect of 4-quinolone-derived molecules on microgametocyte activity.^44^

#### Evaluation of Compound 1 in the Reduction of Exflagellation in an *Ex vivo* Assay

For the *ex vivo* assays, a female CD1 mouse between 4 and 5 weeks of age (n = 3) was infected with *P. berghei.* After 4 days, when the presence of parasites (sexual phases) in the blood was observed by Giemsa-stained blood smear microscopy, the total blood from the mouse was collected into a preheated tube at 37 °C containing RPMI medium supplemented with 20% Fetal Bovine Serum (FBS) and 200 units/mL of heparin as an anticoagulant. The tube was maintained at 37 °C and centrifuged at 1500 rpm for 5 min. The supernatant was discarded, and the blood was resuspended in 20 mL of RPMI medium supplemented with 20% FBS.^45^

One milliliter of resuspended blood was added to each well of a 6-well plate containing 5 μM methylene blue (positive control), test compound 1 at 10 μM and 1 μM, and DMSO (negative control). After 15 min, the wells were homogenized, and 10 μL of the content was collected into an Eppendorf tube and incubated at room temperature for another 15 min. The sample was then transferred to a Neubauer chamber, and exflagellation centers were counted using an optical microscope at 40× magnification.

#### *In Vitro* Activity against Hepatic *Plasmodium berghei* Infection

Huh-7 cells were cultured in RPMI 1640 medium (Sigma) supplemented with 10% v/v fetal bovine serum (FBS), 1% v/v penicillin/streptomycin, 1% v/v glutamine, 1% v/v nonessential amino acids, and 10 mM HEPES, pH 7. For compound dilution, a medium containing 1:1000 gentamicin (G) and 1:300 fungizone (F) was used. Cells were seeded at 1 × 10^4^ cells/well in 96-well plates and incubated at 37 °C with 5% CO_2_. After 24 h, compounds were diluted in RPMI+F+G, and after removal of the culture medium, added to the cells in triplicate. DMSO and atovaquone were used as negative and positive controls, respectively. Following incubation for 1 h at 37 °C, 5% CO_2_, the cells were infected with luciferase-expressing *P. berghei* sporozoites (4,000 parasites per well) freshly extracted from the salivary glands of infected mosquitoes reared at the GIMM insectarium. The viability of Huh-7 cells exposed to the compounds was assessed 46 h later by the AlamarBlue assay (Invitrogen, UK) measurement,^46^ and parasite burden was measured 48 h after sporozoite addition using a bioluminescence assay (Biotium). IC_50_ values were calculated using GraphPad Prism through nonlinear regression analysis of normalized dose–response curves.

#### *In Vitro* Cytotoxicity of 4-Quinolone Compounds in Huh-7 Cells

On the first day of the assay, the compounds were diluted at 10 and 1 μM in cRPMI medium supplemented with fungizone. and gentamicin. The culture medium of Huh-7 cells was removed and replaced with compound dilutions in triplicate. As a control, a DMSO dilution mimicking the highest compound concentration used was included. The cells were incubated for 1 h at 37 °C in a 5% CO_2_ atmosphere.^45^

On the second day of the assay, compound toxicity was assessed by measuring cell confluency using the Alamar Blue/CellTiter Blue reagent. First, reagent aliquots stored at −20 °C were thawed and diluted at a 1:20 ratio in cRPMI. The culture medium was removed and replaced with 80 μL of the solution per well, followed by incubation at 37 °C and 5% CO_2_ for 1 h and 30 min. Fluorescence was measured using a plate reader with the **Tecan i-Control** software. The reading parameters included fluorescence intensity, excitation wavelength at 530 nm and emission at 590 nm, number of flashes set to 10, optimized gain, and the use of a Nucleon 96 flat transparent plate.

#### Mitochondrial Toxicity Assessment of Compound 1 in HepG2 Cells Cultured in Glucose or Galactose

To evaluate mitochondrial toxicity, HepG2 cells were cultured in RPMI-1640 medium under two metabolic conditions: one group was maintained in standard RPMI supplemented with 2 g/L glucose (glucose condition), while the second group was gradually adapted to rely on galactose as the primary energy source. For this adaptation, glucose levels were reduced by 20% every 2 days until reaching a final concentration of 20% of the original glucose amount. On the day of the assay, the glucose-adapted cells were cultured in glucose-free RPMI medium supplemented with 10 mM galactose.^28^ When cells reached 80% confluence, they were detached using 2 mL of trypsin-EDTA solution (0.2 g of EDTA) for 5 min at 37 °C, then resuspended in fresh medium and centrifuged at 100 × g for 1 min. After centrifugation, cells were counted using a Neubauer chamber and seeded at 3 × 10^6^ cells per well in 96-well plates containing 180 μL of culture medium. Plates were incubated at 37 °C with 5% CO_2_ for 12–16 h to allow for cell adhesion. Subsequently, 20 μL of Compound **1** or atovaquone at various concentrations in a serial dilution factor of 2 (ranging from 3.125 to 50 μM) were added, and plates were incubated for 24 h. After treatment, 40 μL of a 0.15 mg/mL resazurin solution (RSZ) was added to each well and incubated for an additional 5 h. RSZ is a nontoxic, cell-permeable blue dye that is reduced by metabolically active cells to highly fluorescent pink resorufin. Fluorescence was measured using a fluorimeter with excitation/emission at 560/590 nm. Cell viability was calculated relative to untreated controls (set at 100%), determined using GraphPad Prism 8.

### Statistical Analysis

Statistical analyses were conducted using GraphPad Prism v0.9.0. The Mann–Whitney test was employed to evaluate the blood-feeding rate and differences in infection prevalence between species. Infection intensity, measured as the average production of oocysts and sporozoites, was analyzed using the same test, considering only mosquitoes with more than zero oocysts. *P. falciparum* asexual forms were also analyzed with GraphPad Prism v0.9.0.^38,46^

## Supplementary Material



## Notes

IC_50_ values of compound **1** in *P. falciparum* strains (Figure S1); IC_50_ values of control compounds in *P. falciparum* *in vitro* assay (Table S1); inhibition of *P. berghei* transmission stages by compound **1** (Table S2); reduction of *P. vivax* oocysts and sporozoites with compounds **1** and **6** (Figure S2); midgut staining of *Anopheles darlingi* with and without treatment (Figure S4); inhibition of *P. berghei* exflagellation centers *in vivo* and *ex vivo* by compound **1** (Figure S5); transmission-reducing activity (TRA) of compound **1** against *P. berghei* oocysts and sporozoites (Figure S6); inhibition of *P. berghei* liver stages and Huh7 cell confluency with 4-quinolone derivatives (Figure S7); chromatogram showing the sequenced region of a *Plasmodium falciparum* sample (Figure S8); sequencing of the PfCYTb gene in *plasmodium falciparum* TM90C6B (Methodology S1); sequencing of the PfCYTb Gene in *Plasmodium falciparum* TM90C6B (Result S1) (PDF)
