# Supplementary material for: Evaluation of the Activity of 4‑Quinolones against Multi-Life Stages of Plasmodium spp
Source: ACS Omega. 2025 Nov 5;10(45):54850–63. doi: 10.1021/acsomega.5c08663 (PMC12631437; doi:10.1021/acsomega.5c08663)
Supplement: Supplementary file 1 [file ao5c08663_si_001.pdf]

Evaluation of the Activity of 4-Quinolones Against Multi-Life Stages of *Plasmodium* spp.

Yasmin Annunciato[1], Everton M. da Silva[2], Jéssica E. Araújo[3], Leandro do N. Martinez[4], Sofia Santana[5], Eyob A. Workneh[5], Luís C. S. Alvarez[6], Marcela L. Magalhães[6], Alice Oliveira Andrade[3], Wallyson de J. da Costa[4] , Guilherme Campolina[7], Matheus Nascimento Santana[7], Camila de S. Barbosa[7], Erica P. M. L. Peres[1] , Caio S. Moura[1], Najara A. C. dos Santos[3], Alessandra da S. Bastos[3], Marcos L. Gazarini[1], Jansen Fernandes Medeiros[3], Carolina B. G. Teles[4], Fabio T. M. Costa[6,8], Ana C. Alves[8], Dhelio B. Pereira[9], João Pinto[8], Pedro V. L. Cravo[8], Rafael Victorio Carvalho Guido[10], Miguel Prudêncio[5,11], Maisa da S. Araujo[3,9], Arlene G. Corrêa[2], Gustavo C. Cassiano[8*], Anna C. C. Aguiar[1,7*]

[1]Universidade Federal de São Paulo, Laboratory of cell biology and biochemistry of parasitic diseases, Biosciences department, Rua Silva Jardim, 136 - Vila Matias, 11015-020, Santos, SP, Brazil.

[2]Universidade Federal de São Carlos, Department of Chemistry, Rodovia Washington Luís, s/n, 13565-905, São Carlos, SP, Brazil.

[3]Fundação Oswaldo Cruz, Malaria Vector Production and Infection Platform (PIVEM)/Entomology Lab, R. da Beira, 7671 – Lagoa, 76812-245, Porto Velho, RO, Brazil.

[4]Fundação Oswaldo Cruz, Bioassay Platform for Malaria and Leishmaniasis, R. da Beira, 7671 – Lagoa , 76812-245, Porto Velho, RO, Brazil.

[5]GIMM - Gulbenkian Institute for Molecular Medicine, Plasmodium infection and anti-malarial interventions, Avenida Prof. Egas Moniz, 1649-035, Lisboa, Portugal.

[6]Laboratory of Tropical Diseases Prof. Luiz Jacintho da Silva, Department of Genetics, Evolution, Microbiology and Immunology, University of Campinas (UNICAMP), Cidade Universitária Zeferino Vaz - Barão Geraldo, 13083-970, Campinas - SP, Brazil

[7]Universidade Federal de São Paulo, Laboratory of Malaria Research, Microbiology and Imunology Departament, Rua Botucatu, 862 - Vila Clementino, 04023-062, São Paulo, SP, Brazil

[8]Global Health and Tropical Medicine, Associate Laboratory in Translation and Innovation Towards Global Health, Instituto de Higiene e Medicina Tropical, Universidade Nova de Lisboa, Rua da Junqueira, 100, 1349-008 Lisbon, Portugal

[9]Centro de Pesquisa em Medicina Tropical de Rondônia - CEPEM, Medicina Tropical e Doenças Infecciosas Department. Av. Guaporé – Lagoa, 415 303, 76.812-329, Porto Velho, RO, Brazil

[10]Universidade de São Paulo, São Carlos Institute of Physics, Avenida Trab. São Carlense, 400 - Parque Arnold Schimidt, 13566-590, São Carlos, SP, Brazil.

[11]Faculdade de Medicina da Universidade de Lisboa, Acadêmia de Ciências da Faculdade de Medicina, Av. Prof. Egas Moniz MB, 1649-028 Lisboa, Portugal

*Universidade Federal de São Paulo, Laboratory of Malaria Research, São Paulo, SP.
annaccaguiar@gmail.com
*Instituto de Higiene e Medicina Tropical, Lisboa, Portugal.
gustavocassiano@ihmt.unl.pt

Supporting Information

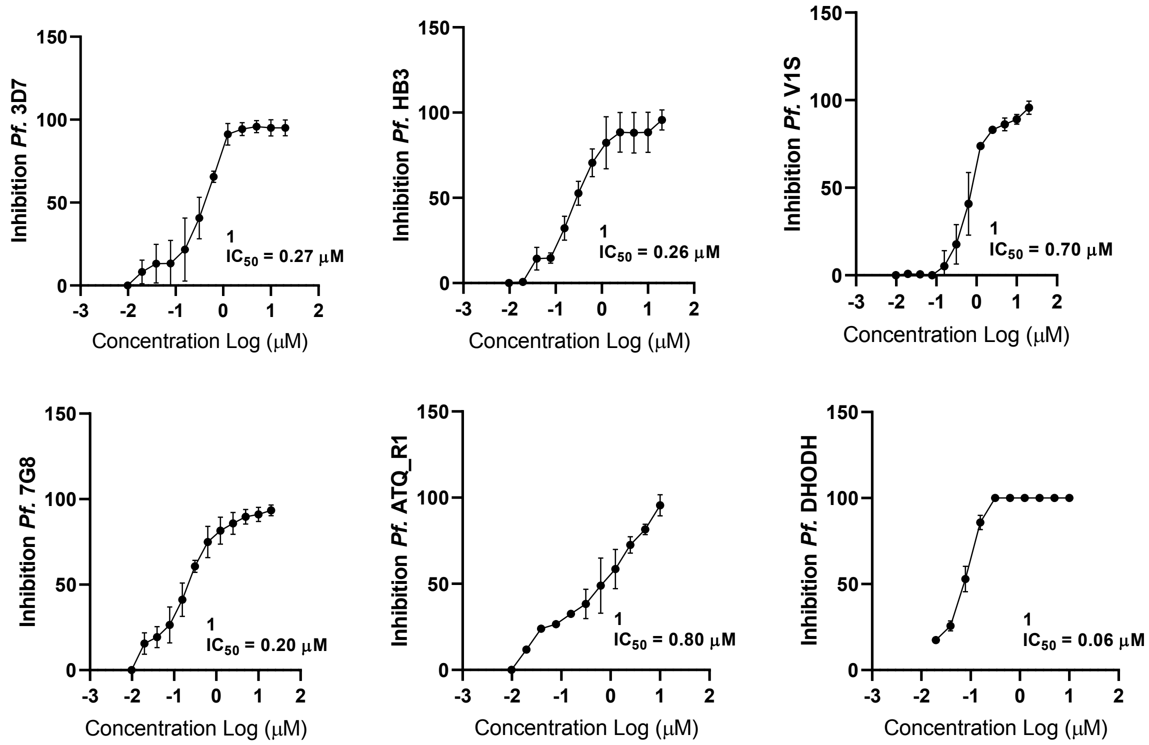

Figure S1. Average IC_{50} of compound **1** in sensitive strain (3D7) and resistant strains (HB3, V1/S, 7G8, ATQ_R1 and DHODH) of *P. falciparum*

Table S1. IC_{50} value of the control compounds of the *P. falciparum in vitro* assay.

	Strains (IC_{50} µM)						
	**3D7**	**HB3**	**V1S**	**7G8**	**SB1-A6**	**ATQ_R1**	**DHODH**
**Chloroquine**	0.006 ± 0.001	0.006 ± 0.002	0.17 ± 0.09	0.22 ± 0.08	-	-	-
**Pyrimethamine**	0.050 ± 0.008	5.2 ± 0.4	6 ± 2.4	6 ± 1	-	>10*	-
**Atovaquone**	0.001 ± 0.0004	0.0007 ± 0.0004	0.009 ± 0.0008	0.006 ± 0.004	>10*	>10*	-
**DSM 265**	-	-	-	-	-	-	>10*

**It was not possible to calculate the IC_{50} due to the high resistance of the tested strains, which exhibited values greater than 10 micromolar for the evaluated compounds.*

Table S2. Inhibition (%) of compound **1** against *P. berghei* exflagelation centers in an *in vivo* and *ex vivo* assay, and oocysts and sporozoites in the *in vivo* assay with *P. berghei*.

	Inhibition Mean (%)					
**Concentration**	**50 mg/kg***		**10 µM**		**5 µM**	**1 µM**
**Compound**	**Prim.**	**1**	**Prim.**	**1**	**MB**	**1**
Exflagelation *P. berghei* (*ex vivo)*	-	-	100	63,90	100	23,80
Exflagelation *P. berghei* (*in vivo)*	100	85,17	-	-	-	-
Oocysts *P. berghei* (*in vivo*)	100	67,00	-	-	-	-
Sporozoites *P. berghei* *(in vivo)*	100	91,30	-	-	-	-

*2 doses of 50 mg/kg spaced over 12 hours was administered for the in vivo assay.

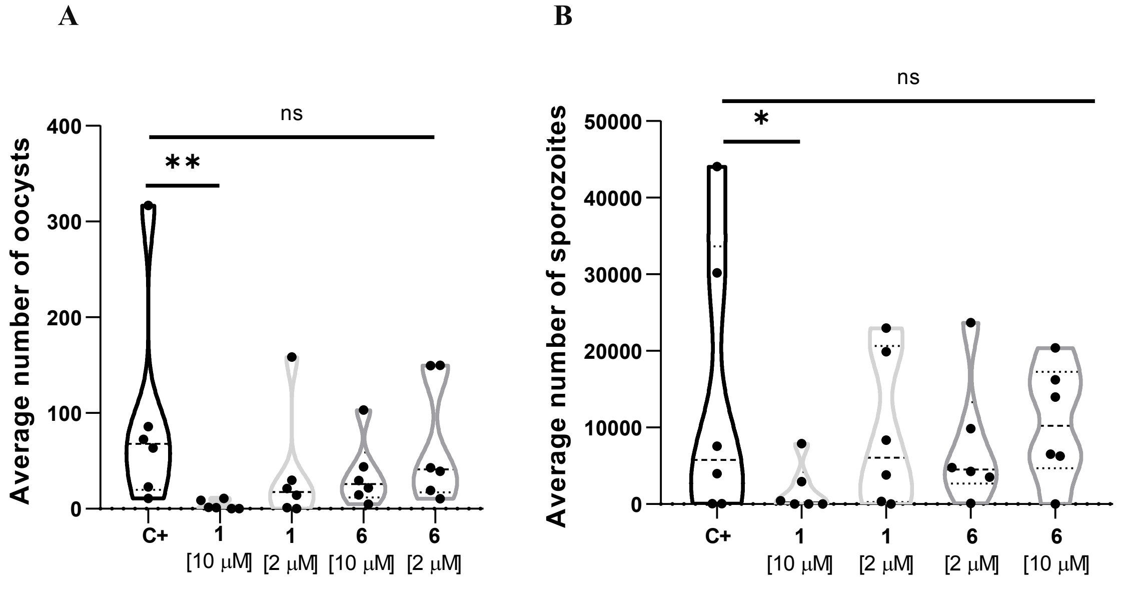

Figure S2. Average reduction of *P. vivax* oocysts in the presence of compounds **1** and **6** tested at 10 µM and 2 µM, respectively, compared to the control group. ** $p = 0.004$; ns = non significative **(A)**. Average reduction of sporozoites in the presence of 4-quinolone derivatives **1** and **6** tested at 10 µM and 2 µM. * $p = 0.004$; ns = non significative **(B)**.

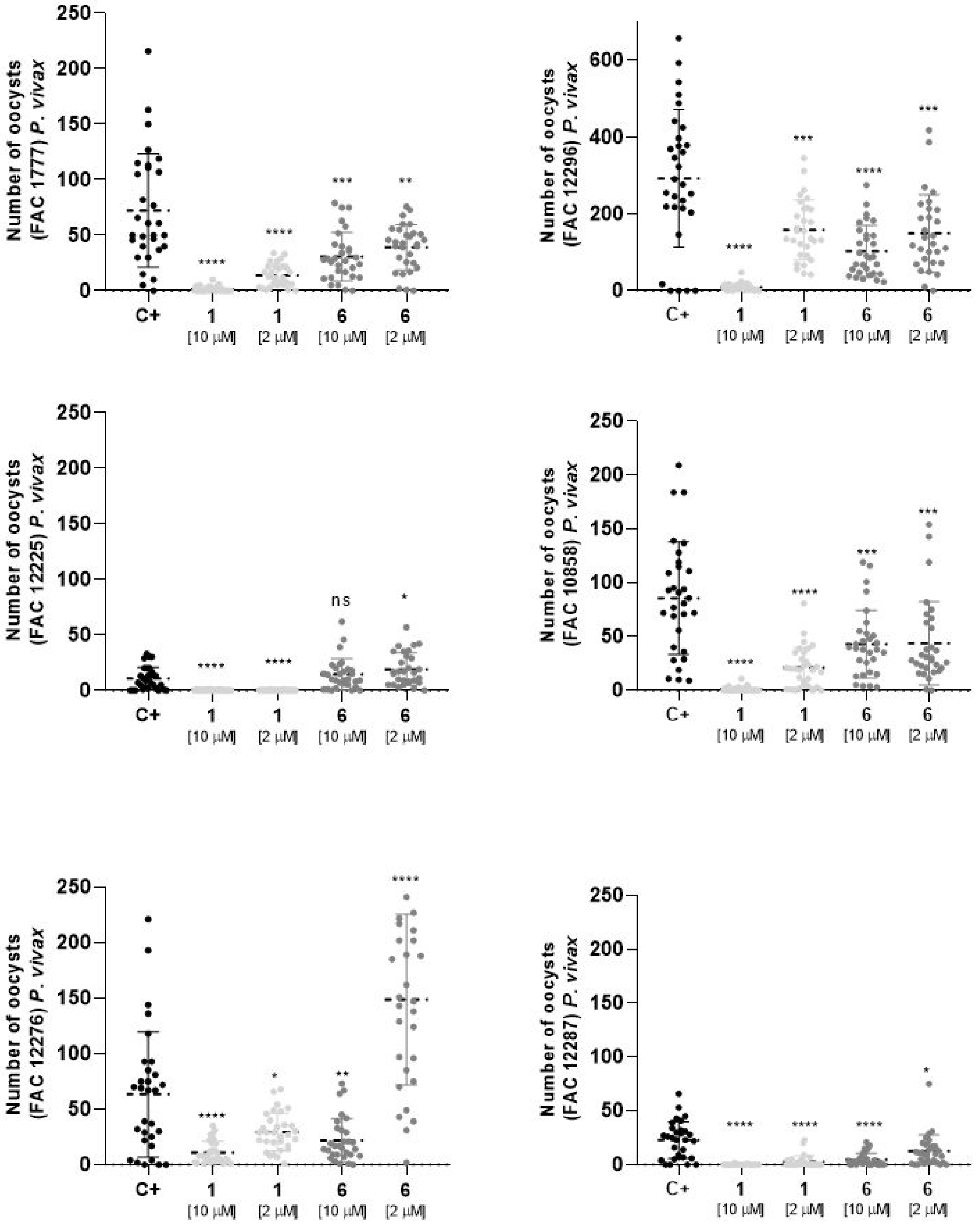

Figure S3. The median of oocysts in 6 different patients (FAC 1777, 12296, 12225, 10858, 12276, 12287) and statistical significance was determined by Mann-Whitney test. **** p<0.0001; ***p = 0,0001; **0,009; *p = 0.01; ns = non-significant.

A **B**

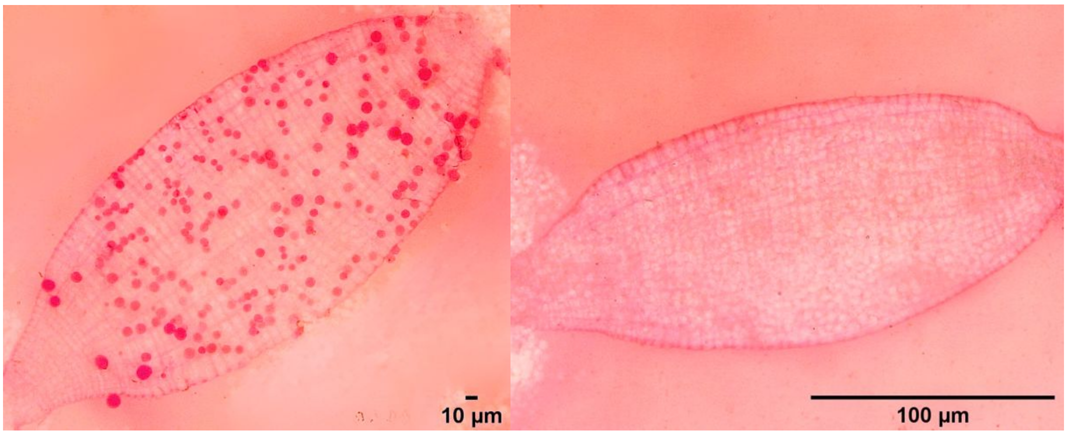

Figure S4. The midgut of *Anopheles darlingi* mosquito from the control group was stained with mercurochrome. The dark pink dots represent lodged oocysts **(A)**. Midgut of the group is treated with compound **1** at 10 µM **(B)**.

A **B**

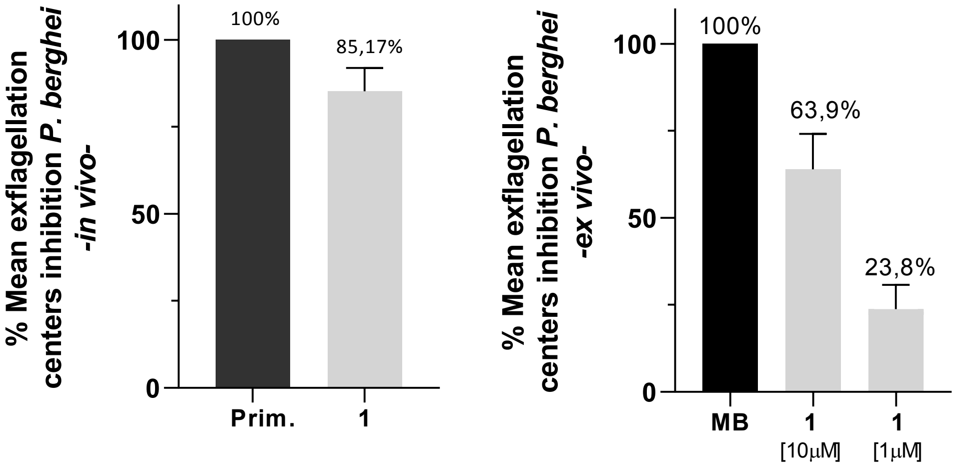

.

Figure S5. Mean of exflagellation centers inhibition of *P. berghei* in an in vivo assay (n=2) **(A)**. Mean of exflagellation centers inhibition of *P. berghei* in an ex vivo assay (n=3). The compound 1 was tested at the concentration of 10 µM and 1 µM **(B)**.

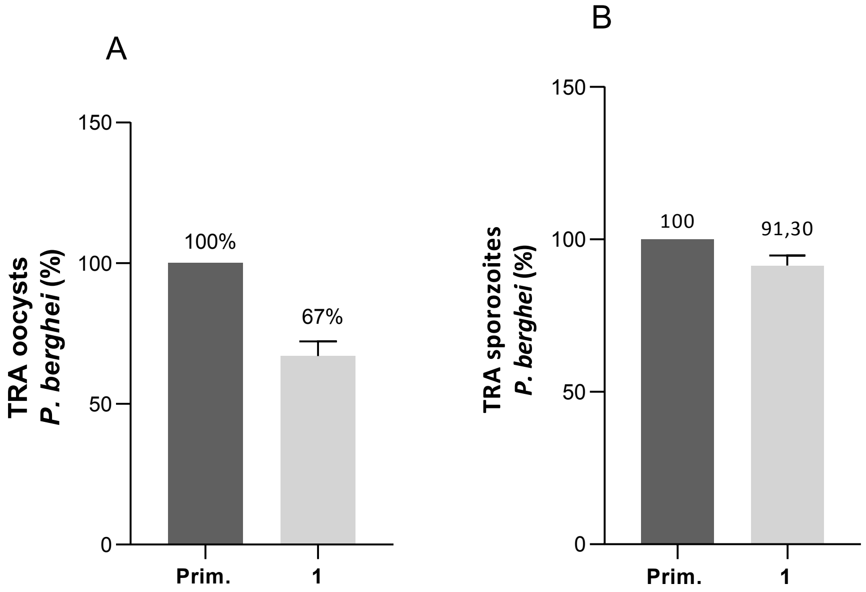

Figure S6. Reduced transmission activity (TRA) of *P. berghei* oocysts in an in vivo assay (n=2) **(A)**. Reduction of transmission activity of *P. berghei* sporozoites **(B)**.

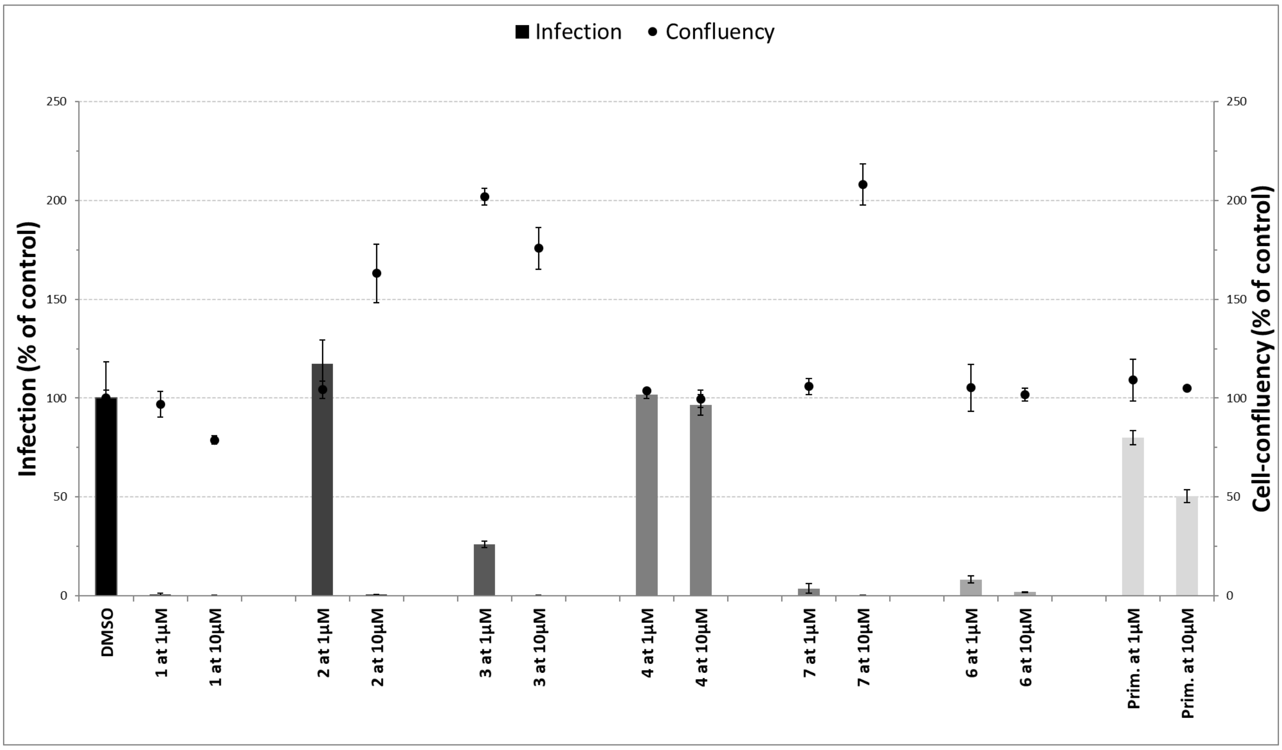

Figure S7. Inhibition mean of *P. berghei* liver stages and the Confluency of Huh7 cells in the presence of the compounds. Compound 5 does not present any inhibition. Black bars: Infection levels (luciferase assay); Red dots: Cell confluency levels (Alamar assay). The data was normalized to DMSO control.

Method S1

Sequencing of the PfCYTb Gene in *Plasmodium falciparum* TM90C6B

Genomic DNA (gDNA) was extracted from 200 µL of TM90C6B-infected blood using the PureLink Genomic DNA Mini Kit (Invitrogen), according to the manufacturer's protocol. The target for amplification was a 934 bp fragment of the mitochondrial *Pf*CYTb gene, which includes codons associated with atovaquone resistance. Primers were designed based on the *P. falciparum* DD2 reference sequence (PlasmoDB ID: PfDd2_000011300)[S1], as follows: forward primer 5′-CATGTCCATTGAACATAAACTT-3′ and reverse primer 5′-GCATAGAATGCACACATAAA-3′.

PCR reactions were prepared in 25 µL volumes containing 1 µL of genomic DNA, 400 nM of each primer, and 2× PCR Biosystems reaction buffer. Amplifications were performed on a BIO-RAD thermal cycler under the following conditions: initial denaturation at 95 °C for 5 minutes; followed by 35 cycles of denaturation at 95 °C for 30 seconds, annealing at 55 °C for 30 seconds, and extension at 60 °C for 2 minutes; with a final extension at 65 °C for 5 minutes[S2].

PCR products were resolved on 2% agarose gels stained with ethidium bromide and visualized under UV light. The expected bands were excised and purified using the PureLink Quick Gel Extraction Kit (Thermo Fisher Scientific). Purified amplicons were subjected to Sanger sequencing using the BigDye Terminator v3.1 Cycle Sequencing Kit and the ABI Prism 3730 DNA Analyzer (Applied Biosystems).

Sequencing reads from both directions were aligned using BioEdit Sequence Alignment Editor version 7.2.5. The resulting consensus sequence was compared to the DD2 reference, and the presence of the Y268S mutation (TAT → TCT) in the *Pf*CYTb gene was confirmed by visual inspection of the chromatograms. No mutations were detected at codon 272. The Y268S mutation is known to confer resistance to atovaquone by impairing the function of mitochondrial complex III, its primary molecular target.

Structure modeled using AlphaFold (AF-Q6PPF5-F1) and visualized in ChimeraX, with mutated residues highlighted in color.

Result S1

The sequencing confirmed the presence of the Y268S mutation (codon change TAT → TCT) in the mitochondrial *Pf*CYTb gene of the *Plasmodium falciparum* TM90C6B strain. This point mutation is well-characterized and known to confer high-level resistance to atovaquone by altering the Qo binding site of the cytochrome *b* subunit within the bc1 complex, thereby impairing the drug's ability to inhibit mitochondrial electron transport. Importantly, no additional mutations were detected at codon 272, suggesting that the resistance phenotype is primarily associated with the Y268S substitution.

The confirmation of this mutation is particularly significant in the context of our study, as Compound **1** demonstrated potent activity against this resistant strain. Since Compound **1** also targets the bc1 complex of *P. falciparum*, its ability to inhibit parasite growth despite the presence of the Y268S mutation suggests a distinct binding mode or interaction within the complex, possibly circumventing the resistance mechanism that renders atovaquone ineffective. This highlights the potential of Compound **1** as a promising therapeutic candidate

for the treatment of malaria caused by atovaquone-resistant strains and underscores the value of developing mitochondrial-targeting agents with alternative resistance profiles.

Amplicon:

F: 5’-CATGTCCATTGAACATAAACTT-3’

R: 5’-GCATAGAATGCACACATAAA-3’

5’-
CATGTCCATTGAACATAAACTTTTTATGGAATTACGGATTCCTTTTAGGAATAATATTTTT
TATTCAAATTATAACAGGTGTATTTTTAGCAAGTCGATATACACCAGATGTTTCATATGC
ATATTATAGTATACAACACATTTTAAGAGAATTATGGAGTGGATGGTGTTTTAGATACAT
GCACGCAACAGGTGCTTCTCTTGTATTTTTATTAACATATCTTCATATTTTAAGAGGATTA
AATTACTCATATATGTATTTACCATTATCATGGATATCTGGATTGATTTTATTTATGATAT
TTATTGTAACTGCTTTCGTTGGTTATGTCTTACCATGGGGTCAAATGAGTTATTGGGGTGC
AACTGTAATTACTAACTTGTTATCCTCTATTCCAGTAGCAGTAATTTGGATATGTGGAGG
ATATACTGTGAGTGATCCTACAATAAAACGATTTTTTGTACTACATTTTATCTTACCATTT
ATTGGATTATGTATTGTATTTATACATATATTTTTCTTACATTTACATGGTAGCACAAATC
CTTTAGGGTATGATACAGCATTAAAAATACCCTTTTATCCAAATCTATTAAGTCTTGATGT
TAAAGGATTTAATAATGTTATAATTTTATTTCTAATACAAAGTTTATTTGGAATTATACCT
TTATCACATCCTGATAATGCTATCGTAGTAAATACATATGTTACTCCATCTCAAATTGTAC
CTGAATGGTACTTTCTACCATTT**TAT**GCAATGTTA**AAA**ACTGTTCCAAGTAAACCAGCTG
GTTTAGTAATTGTATTATTATCATTACAATTATTATTCTTATTAGCAGAACAAAGAAGTTT
AACAACTATAATTCAATTTAAAATGATTTTTGGTGCTAGAGATTATTCTGTTCCTATTATA
TGGTTTATGTGTGCATTCTATGC-3’ (934pb)

TAT – Mutation in Y268S

AAA – Mutation in K272R

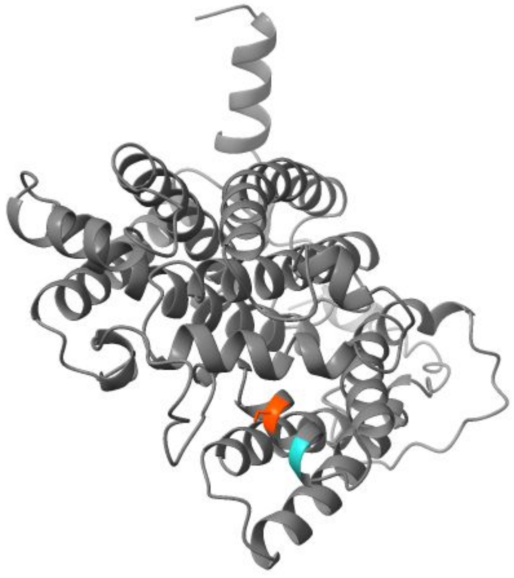

Figure S8. 3D structure of the *P. falciparum* cytochrome *b* from Alpha Fold (AF-Q6PPF5-F1), with mutations associated with atovaquone resistance: The **Y268S** mutation is shown in

orange, a substitution at the catalytic site (tyrosine to serine) that interferes with atovaquone binding and is a classically substituted in atovaquone-resistant parasites. The **K272R** mutation is shown in blue, a lysine-to-arginine substitution near the quinol binding pocket, potentially affecting drug interaction and protein stability.

References

(S1) *Plasmodium falciparum* DD2 cytochrome b gene (PfDd2_000011300); PlasmoDB: https://plasmodb.org/plasmo/app/record/gene/PfDd2_000011300 (accessed June, 02, 2025).

(S2) Srivastava, I. K.; Morrisey, J. M.; Darrouzet, E.; Daldal, F.; Vaidya, A. B. Resistance mutations reveal the atovaquone-binding domain of *Plasmodium falciparum* cytochrome b. *Mol. Microbiol.* 1999, *33* (4), 704–711. https://doi.org/10.1046/j.1365-2958.1999.01520.x. (PMCID: PMC4354981)
